# Per- and Polyfluoroalkyl Substances (PFAS) in Community Water Systems (CWS) and the Risk of Thyroid Cancer: An Ecological Study

**DOI:** 10.3390/toxics11090786

**Published:** 2023-09-16

**Authors:** Mathilda Alsen, Angela M. Leung, Maaike van Gerwen

**Affiliations:** 1 Department of Otolaryngology-Head and Neck Surgery, Icahn School of Medicine at Mount Sinai, New York, NY 10029, USA; 2 Division of Endocrinology, Diabetes, and Metabolism, Department of Medicine, David Geffen School of Medicine at University of California Los Angeles, Veterans Affairs Greater Los Angeles Healthcare System, Los Angeles, CA 90024, USA; 3 Institute for Translational Epidemiology, Icahn School of Medicine at Mount Sinai, New York, NY 10029, USA

**Keywords:** endocrine-disruptive chemicals, EDCs, thyroid cancer, per- and polyfluoroalkyl substances, PFAS, perfluorononanoic acid, PFNA, perfluorooctanoic acid, PFOA, epidemiology

## Abstract

Thyroid cancer incidence has been steadily increasing over the past decade in the United States (US). A discussion exists regarding the potential contribution of exposure to endocrine-disrupting chemicals, encompassing certain per- and poly-fluoroalkyl substances (PFASs). This ecological study evaluated the potential correlation between PFAS levels in drinking water and thyroid cancer incidence in the US. Data on age-adjusted thyroid cancer incidence rate (per 100,000 persons) by county were obtained from the Centers for Disease Control and Prevention (CDC) for US counties with available data in 2015–2019. Data on PFAS concentrations in the drinking water of selected community water systems (CWSs) were obtained from the CDC National Environmental Public Health Tracking Network in 2013–2015. The correlation between PFASs in CWSs and thyroid cancer incidence was calculated using Spearman correlation. A statistically significant correlation was found between perfluorooctanoic acid (PFOA) (r = 0.031; *p* = 0.043), perfluorononanoic acid (PFNA) (r = 0.058; *p* ≤ 0.001), and thyroid cancer incidence. The results suggest a potential link between certain PFAS exposures and thyroid cancer risk. However, due to the nature of an ecological study, no conclusions can be drawn at the individual level or causality. More research is needed, particularly on an individual level to allow for more detailed exposure assessment.

## 1. Introduction

The incidence rate of thyroid cancer has increased markedly in recent decades in the United States (US) and worldwide [1]. This increase may partially be a reflection of overdiagnosis due to the implementation of more frequent screenings, as well as the rise in neck ultrasonography capturing small tumors [2]. Although overdiagnosis may partly explain the increased incidence, recent studies have highlighted environmental exposures, particularly endocrine-disruptive chemicals (EDCs), as potential contributors [3,4]. Endocrine-disruptive chemicals (EDCs) may alter thyroid function and have been associated with some cancer types, including testicular and prostate cancer [4]. Per- and polyfluoroalkyl substances (PFASs), a group of persistent EDCs, have become a growing concern in recent years due to increasing evidence of potentially causing adverse health effects, including liver damage, pregnancy-induced hypertension, decreased response to vaccines, and developmental effects, including decreased birth weight [5]. 

PFASs are synthetic chemicals introduced in the 1940s and used for commercial manufacturing. PFASs can be found in everyday products, including food containers and packaging, clothing, cleaning products, and personal care products [6]. The main source of exposure to PFASs is through contaminated drinking water and food [6,7,8]. Different PFASs are often found in drinking water [7]. There are currently nearly 15,000 different types of PFASs identified, and the most common are perfluorooctanoic acid (PFOA), perfluorooctanoic sulfonic acid (PFOS), and perfluorononanoic acid (PFNA) [9]. The serum elimination half-life for PFASs is several years: about 3 years for PFOA and 4 years for PFOS [10]. PFASs have been detected in the serum of 97% of US individuals [11]. Although the manufacturing of PFOA and PFOS was discontinued in the early 2000s in the US, PFASs can still be found in the environment due to their resistance and stable chemical structure. In 2021, the US Environmental Protection Agency (EPA) identified the concern for PFASs, which was followed by recent rules to establish maximum contaminant levels (MCLs) for six PFASs [12]. 

The International Agency for Research on Cancer (IARC) has classified PFOA, the most researched PFAS, as a possible human carcinogen [13]. A review of epidemiological evidence linking PFASs to cancer was conclusive for testicular and kidney cancer, and a potential association with prostate cancer was found in some studies [14]. Vieira et al. examined cancer cases among residents by the DuPont Teflon-manufacturing plant and reported that PFOA may be associated with testicular, kidney, prostate, and ovarian cancers and non-Hodgkin lymphoma [15]. As it relates to thyroid cancer, Barry et al. found a positive but non-significant association between exposure to PFOA and hazard of thyroid cancer among 32,254 PFOA-exposed participants (HR: 1.10; 95% CI: 0.95–1.26) [16]. Recently, van Gerwen et al. were the first to find a significantly positive association between linear PFOS and thyroid cancer when comparing the plasma of 88 thyroid cancer patients with 88 non-cancer controls (odds ratio (OR): 1.56; 95% confidence interval (CI): 1.17–2.15) [17]. However, a case–control study in China demonstrated that higher levels of the PFASs PFNA, PFDA, and PFUnDA as well as PFAS mixtures were associated with a lower risk of thyroid cancer, but since the study participants were recruited from the same hospital, the results may not be generalizable to the general population [18]. 

Given the population’s exposure to PFAS and the potential carcinogenic effects of PFAS exposure on the thyroid gland, this ecological study evaluated the potential correlation between PFAS exposure in drinking water and thyroid cancer incidence by county in the US. 

## 2. Materials and Methods 

Age-adjusted thyroid cancer incidence rates (per 100,000 persons) by county were obtained from the Centers for Disease Control and Prevention (CDC) for US counties for 2015–2019, as county-level data are only available for a 5-year period. The incidence data have been compiled from selected cancer registries meeting US Cancer Statistics data quality criteria, covering 97% of the US population [19]. Age-adjusted thyroid cancer incidence rates (per 100,000 persons) by sex were also obtained from the CDC for US counties. Smoothed data were used for the sex-specific data, where the data borrow from neighboring areas to stabilize results, particularly in areas where population is sparse [20]. 

PFAS levels in drinking water for selected community water systems (CWSs) were obtained from the CDC National Environmental Public Health Tracking Network [20]. According to the CDC, these levels were obtained from the US EPA’s National Contaminant Occurrence Database sampling data for six different PFASs between January 2013 and December 2015. The selected CWSs were required to sample the water systems once, sample twice if the water source was groundwater, and sample four times if the water source was surface water; however, not all CWSs were required to sample their water. These sampling measures were created following the US EPA Third Unregulated Contaminant Monitoring Rule (UCMR3), a program that collects data for contaminants in drinking water that do not have health-based standards set under the Safe Drinking Act (SDWA) [20,21]. The limits of detection were based on the minimum reporting limits (µg/L) as defined by UCMR3 for the six PFASs, which included perfluorooctanoic acid (PFOA), perfluorononanoic acid (PFNA), perfluorooctane sulfonic acid (PFOS), perfluorohexane sulfonate (PFHxS), perfluoroheptanoic acid (PFHpA), and perfluorobutane sulfonate (PFBS) with minimum reporting limits (MRL) (µg/L) of −0.02, −0.02, −0.04, −0.03, −0.01, and −0.03, respectively [22]. 

For values at or less than the MRL for each PFAS, we assigned a value equal to the MRL divided by the square root of two, following NHANES recommendations. This value was assigned to address non-zero values below the MRL [23]. 

The data were imported into the mapping software ArcGIS, developed by the Environmental Systems Research Institute (version 10:8; ESRI, Redlands, CA, USA). For visual purposes, the maps display thyroid cancer incidence by state between 2015 and 2019 due to a lack of thyroid cancer data for some counties. To examine the possible link between PFAS exposure and thyroid cancer incidence risk, we calculated the correlation between PFAS levels for each CWS and the thyroid cancer incidence rate for the county of the CWS. Since the data do not have a normal distribution, the Spearman correlation coefficient was used. The 1634 counties, out of 3142, with missing data on thyroid cancer incidence rates were excluded from the analysis. Spearman correlation between PFAS levels (µg/L) and thyroid cancer incidence rates were performed using the R software (version 4. 1.2; Vienna, Austria). 

## 3. Results 

**Figure 1:**
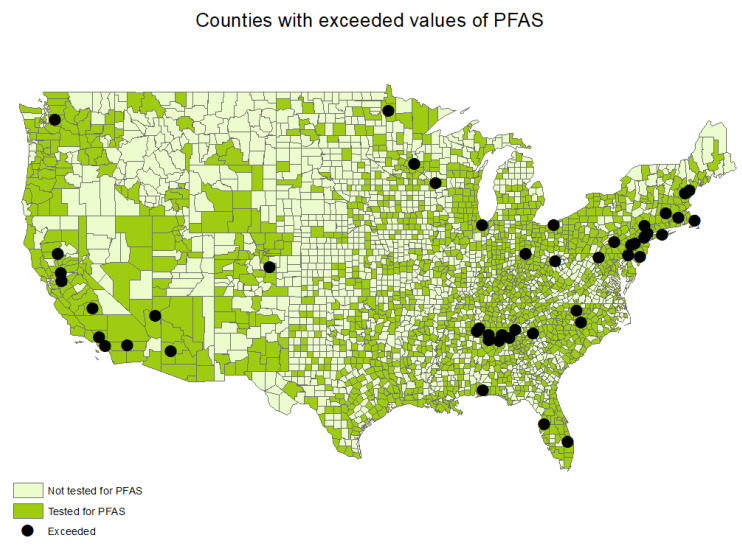
Counties with CWSs sampled and exceeded values of PFASs.

Approximately 50% of all US counties had at least one CWS sampled for PFASs (*n* = 1605) between the years 2013 and 2015. (Table 1, Figure 1). The state with the most CWSs sampled for PFASs was California (*n* = 455), followed by Texas (*n* = 386) and Florida (*n* = 262) (Table 1). All US states had at least one CWS sampled for PFASs, and the selected CWSs were tested for all six PFASs. A total of 4795 CWSs were sampled for PFASs between the years 2013 and 1015. Among those, 1.3% of all CWSs that were sampled for PFASs reported exceeding PFAS values (*n* = 63), and 7% of all CWSs that were sampled for PFASs reported detected PFAS values (*n* = 353). 

**Figure 2:**
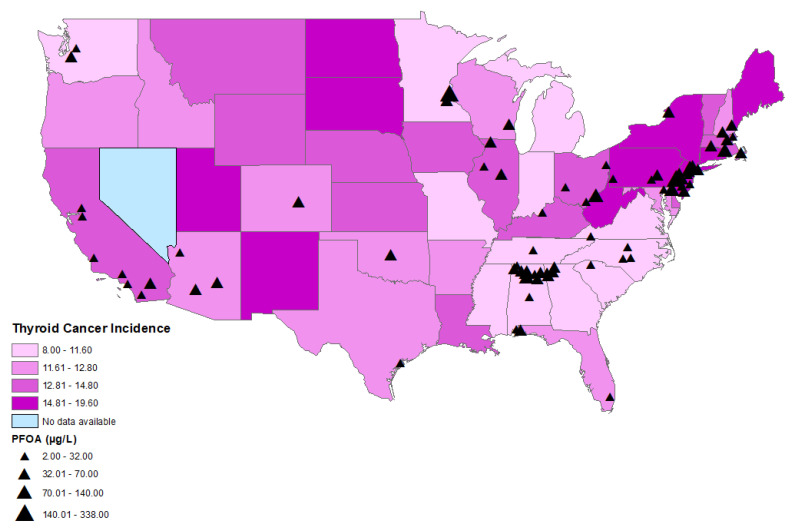
Distributions of age-adjusted thyroid cancer incidence per state and locations of CWSs with detections of PFOA.

**Figure 3:**
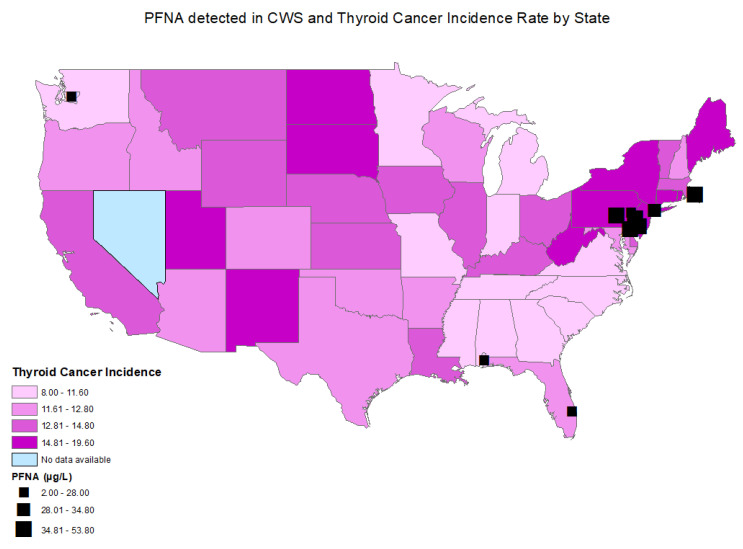
Distributions of age-adjusted thyroid cancer incidence per state and locations of CWSs with detections of PFNA.

The age-adjusted thyroid cancer incidence rates between 2015 and 2019 by county ranged from 5.5 thyroid cancer cases per 100,000 persons in Bowie, Texas to 36.0 thyroid cancer cases per 100,000 persons in Powell, Kentucky. The statewide age-adjusted thyroid cancer incidence rate in 2019 ranged from 8.0 to 19.6 cases per 100,000 persons in Mississippi and in New York, respectively (Figure 2 and Figure 3). Thyroid cancer incidence rate was not available in the state of Nevada, as it did not meet the US Cancer Statistics publication criteria [24].

A statistically significant correlation was found between perfluorooctanoic acid (PFOA) (r = 0.031; *p* = 0.043) (Figure 2, Table 2), perfluorononanoic acid (PFNA) (r = 0.058; *p* ≤ 0.001), and thyroid cancer incidence by county (Figure 3, Table 2). There was no statistically significant correlation between perfluorooctane sulfonic acid (PFOS) (r = −0.016; *p* = 0.292), perfluorohexane sulfonate (PFHxS) (r = −0.003; *p* = 0.827), perfluoroheptanoic acid (PFHpA) (r = 0.004; *p* = 0.789), or perfluorobutane sulfonate (PFBS) (r = −0.028; *p* = 0.069) and thyroid cancer incidence by county. 

Among males, a statistically significant correlation was found between perfluorooctanoic acid (PFOA) (r = 0.071; *p* = 0.009) (Table 3), perfluorononanoic acid (PFNA) (r = 0.098; *p* ≤ 0.001), and thyroid cancer incidence by county (Table 3). There was no statistically significant correlation between perfluorooctane sulfonic acid (PFOS) (r = −0.002; *p* = 0.952), perfluorohexane sulfonate (PFHxS) (r = −0.032; *p* = 0.246), perfluoroheptanoic acid (PFHpA) (r = −0.009; *p* = 0.752), or perfluorobutane sulfonate (PFBS) (r = −0.006; *p* = 0.835) and thyroid cancer incidence by county.

Among females, a statistically significant correlation was found between perfluorooctanoic acid (PFOA) (r = 0.064; *p* = 0.015) (Table 3), perfluorononanoic acid (PFNA) (r = 0.096; *p* ≤ 0.001), and thyroid cancer incidence by county (Table 3). There was no statistically significant correlation between perfluorooctane sulfonic acid (PFOS) (r = −0.015; *p* = 0.573), perfluorohexane sulfonate (PFHxS) (r = −0.042; *p* = 0.115), perfluoroheptanoic acid (PFHpA) (r = −0.021; *p* = 0.417), or perfluorobutane sulfonate (PFBS) (r = −0.023; *p* = 0.334) and thyroid cancer incidence by county. 

CWSs with elevated PFOA levels were mainly located on the East Coast in states with high age-adjusted thyroid cancer incidence, including Pennsylvania, New Jersey, New York, and Delaware (Figure 2). There was a cluster of CWSs with elevated PFOA levels in the state of Alabama, but with a lower age-adjusted thyroid cancer incidence (Figure 2). 

Elevated PFNA levels were also detected mainly on the East Coast, including in Pennsylvania, New York, New Jersey, Delaware, and Rhode Island, which are states with a high age-adjusted thyroid cancer incidence. (Figure 3) 

## 4. Discussion

The present ecological study showed a significant correlation between PFNA and PFOA levels in CWSs and age-adjusted thyroid cancer incidence in US counties. Elevated levels of PFNA and PFOA were predominantly detected in CWSs in Northeastern states with high thyroid cancer rates, including Pennsylvania, Delaware, Rhode Island, New Jersey, and New York. These results suggest that certain PFASs may be associated with thyroid cancer risk, warranting increased surveillance of residents in areas of elevated PFAS exposure. 

Due to increasing concerns about the health effects of PFASs, there have been rapid regulatory developments in the US. In March 2023, the EPA proposed a National Primary Drinking Water Regulation (NPDWR) to establish maximum contaminant levels (MCLs) for PFOA and PFOS [25]. These proposed MCLs of 4 ppt for each PFAS will be legally enforceable levels of PFASs detected in drinking water. PFNA, PFHxS, PFBS, and HFPO-DA will be regulated as a mixture where the water systems would use a hazard index calculation, to determine if a combination of the PFASs could cause any potential risk. The proposed regulation would require public water systems to monitor for PFASs, notify the public of the level of PFASs, and reduce the levels of PFASs if the level would exceed the standards [7,25]. In Europe, PFASs have been regulated for more than a decade. Since 2009, PFOS has been included in the international Stockholm Convention in order to eliminate its use, and PFOA was banned in 2020. In June 2022, PFHxS was added to the ban, which will take effect at the end of 2023. In 2021, the limit for all PFASs in drinking water was set to 0.5 µg/L in Europe [26]. These recent actions for policy change in the US and Europe suggest an increased concern, highlighting the urgent need to fully understand the long-term health effects of PFAS exposure and the possible link to thyroid cancer risk. 

Thyroid cancer is the most common endocrine cancer [27]. Currently, the only established environmental risk factor for thyroid cancer is exposure to ionizing radiation [28]. Other environmental exposures, such as certain pesticides, polychlorinated biphenyls (PCBs), flame retardants, and phthalates, have been shown to interfere with endocrine signaling at the cellular and molecular levels and with thyroid dysfunction [3]. Exposure to some heavy metals with carcinogenic properties has been known to accumulate in the thyroid more than other tissues and may affect the homeostatic mechanisms of the thyroid gland [29]. In addition, different environmental factors, including EDCs, may act on the thyroid through combined mechanisms and with trans-generational effects, which may contribute to the risk of thyroid cancer. However, the role of PFASs in thyroid carcinogenesis and cancer progression is currently unclear. One study found strong evidence that exposure to multiple PFASs induces oxidative stress, immunosuppression, and modulates receptor-medicated effects as potential carcinogenic pathways [3,30]. It has been suggested that long-chain PFASs (PFOA, PFHxS, PFNA) may increase reactive oxygen species (ROS) [29]. Oxidative stress is an imbalance between the productions of ROS [30]. There are not enough data to assess whether PFASs promote chronic inflammation, cellular immortalization, or altering DNA repair. However, the peroxisome proliferator-activated receptors may be activated after exposure to certain PFASs, which is a known component in the development of thyroid cancer [30]. In addition, PFASs compete at thyroid hormone binding sites and induce T4 glucurunidation following excretion, which may result in the disruption of thyroid hormone physiology [3].

While the link between PFAS exposure and thyroid cancer remains unclear, some studies have reported on the association between PFAS exposure and thyroid disruption. One study using the National Health and Nutrition Examination Survey (NHANES) found that a higher concentration of serum PFOA and PFOS was associated with thyroid diseases in the US adult population [31]. A meta-analysis by Kim et al. concluded that exposure to PFASs is negatively associated with total T4 levels, depending on the PFAS concentration [32]. The impact of PFAS exposure on the thyroid gland and carcinogenesis remains an important area of research, especially given the globally rising thyroid cancer incidence trends [33]. 

The reported cluster of high thyroid cancer incidence rates in the northeast area of the US has limited research to date. One study reported that thyroid cancer increased at a faster rate in Pennsylvania than the rest of the country, possibly because of the Three Mile Island nuclear accident, which occurred in 1979 [34]. However, other studies have not been able to confirm this hypothesis [35,36,37,38]. In the current study, Pennsylvania had the highest value of PFNA among all states. In January 2023, Pennsylvania State set regulatory limits for PFASs in drinking water to 14 ppt for PFOA and 18 ppt for PFOS due to the growing health concerns of PFAS exposure. 

This study has several limitations. Due to the nature of ecological studies, the current study cannot link PFAS exposure and thyroid cancer risk at an individual level, as information about individual exposure to PFASs from CWSs was lacking. Furthermore, adjustment for potential important confounding variables such as race–ethnicity and insurance status was not possible due to the ecological study design. In addition, thyroid cancer incidence and PFAS sampling data were missing for numerous counties, which may be due to the data being suppressed, meaning there were fewer than 16 cases reported per category. It may be beneficial for future studies to look at specific states with sufficient data for each county, particularly in the Northeastern states. There was a significant correlation between PFNA and PFOA levels and age-adjusted thyroid cancer incidence among both males and females. However, the data used for this analysis were smoothed, and should be interpreted as a pattern rather than results. Lastly, temporality between exposure and thyroid cancer diagnosis could not be assessed, and the potential period between PFAS exposure data collection (2013–2015) and thyroid cancer incidence (2015–2019) was likely too short to account for latency. To the best of our knowledge, this is the first ecological study investigating PFAS exposure in CWSs and thyroid cancer risk using publicly available data, which consisted of PFAS levels in counties throughout the US. This study thus provides important information about the potential link between PFAS exposure and thyroid cancer risk. Due to increasing evidence of adverse health risks, future longitudinal, prospective studies are needed to investigate different types of PFASs and their role in thyroid cancer development. Future studies should additionally investigate exposure to PFASs during critical development periods in humans, as exposure to some PFASs, depending on the time of exposure in life, may increase susceptibly for chronic health conditions later in life [39]. 

## 5. Conclusions

The present ecological study showed a significant correlation between PFNA and PFOA levels in CWSs and age-adjusted thyroid cancer incidence in US counties. The elevated PFAS levels were predominantly detected in Northeastern states with high thyroid cancer rates. These results warrant increased PFAS monitoring among residents in these areas and inform future studies investigating the potential effects of this exposure on the thyroid gland. However, due to the nature of an ecological study, no conclusions can be drawn at the individual level or causality. More research is needed, particularly on an individual level, to allow for more detailed exposure assessment.

## Figures and Tables

**Table 1:** CWSs sampled for PFASs in each state.

State	CWSs Sampled (n)	CWSs with PFASs Detected (n)	CWSs with Detected PFASs (%)	CWS with Exceeded PFASs (n)	CWS with Exceeded PFASs (%)
Alabama	124	36	29	9	7
Alaska	10	0	-	0	-
Arizona	74	16	22	3	4
Arkansas	63	0	-	0	-
California	455	48	11	10	2
Colorado	81	13	16	3	4
Connecticut	42	0	-	0	-
Delaware	13	7	54	1	8
District of Columbia	3	0	-	0	-
Florida	262	23	9	3	1
Georgia	128	13	10	2	2
Hawaii	20	0	-	0	-
Idaho	26	0	-	0	-
Illinois	256	5	2	1	0.3
Indiana	103	2	2	1	0.9
Iowa	58	0	-	0	-
Kansas	45	1	2	0	-
Kentucky	121	2	2	0	-
Louisiana	89	0	-	0	-
Maine	18	3	17	1	6
Maryland	39	1	3	0	-
Massachusetts	170	14	8	2	1
Michigan	161	2	1	0	-
Minnesota	98	12	12	2	2
Mississippi	77	0	-	0	-
Missouri	87	0	-	0	-
Montana	15	0	-	0	-
Nebraska	21	0	-	0	-
Nevada	18	0	-	0	-
New Hampshire	23	5	22	1	4
New Jersey	175	36	21	6	3
New Mexico	30	1	3	0	-
New York	170	16	9	4	2
North Carolina	151	27	18	2	1
North Dakota	13	0	-	0	-
Ohio	186	10	5	2	1
Oklahoma	67	2	3	0	-
Oregon	65	0	-	0	-
Pennsylvania	178	27	15	5	3
Rhode Island	17	2	12	1	6
South Carolina	82	2	2	0	-
South Dakota	18	1	6	0	-
Tennessee	136	2	1	0	-
Texas	386	4	1	0	-
Utah	62	0	-	0	-
Vermont	12	0	-	0	-
Virginia	78	2	3	0	-
Washington	132	8	6	1	0.8
West Virginia	32	6	19	2	6
Wisconsin	92	4	4	1	1
Wyoming	12	0	-	0	-

**Table 2:** PFAS levels (2013–2015) correlated with county-level thyroid cancer incidence rates (2015–2019).

PFAS	Correlation Coefficient (r)	*p*-Value
perfluorooctanoic acid (PFOA)	0.031	0.043
perfluorononanoic acid (PFNA)	0.058	<0.001
perfluorooctane sulfonic acid (PFOS)	−0.016	0.292
perfluorohexane sulfonate (PFHxS)	−0.003	0.827
perfluoroheptanoic acid (PFHpA)	0.004	0.789
perfluorobutane sulfonate (PFBS)	−0.028	0.069

**Table 3:** PFAS levels (2013–2015) correlated with county-level thyroid cancer incidence rates (2015–2019) by sex. (Smoothed data).

PFAS	Correlation Coefficient (r) Male	*p*-Value Male	Correlation Coefficient (r) Female	*p*-Value Female
perfluorooctanoic acid (PFOA)	0.071	0.009	0.064	0.015
perfluorononanoic acid (PFNA)	0.098	<0.001	0.096	<0.001
perfluorooctane sulfonic acid (PFOS)	−0.002	0.952	0.015	0.573
perfluorohexane sulfonate (PFHxS)	0.032	0.246	0.042	0.115
perfluoroheptanoic acid (PFHpA)	−0.009	0.752	0.021	0.417
perfluorobutane sulfonate (PFBS)	−0.006	0.835	−0.023	0.334

## Data Availability

Publicly available datasets were analyzed in this study. These data can be found here: https://ephtracking.cdc.gov/DataExplorer/ (accessed on 18 August 2023) and https://gis.cdc.gov/Cancer/USCS/#/AtAGlance/ (accessed on 18 August 2023).
